# Increased tartrate-resistant acid phosphatase (TRAP) expression in malignant breast, ovarian and melanoma tissue: an investigational study

**DOI:** 10.1186/1471-2407-6-199

**Published:** 2006-07-25

**Authors:** A Honig, L Rieger, M Kapp, M Krockenberger, M Eck, J Dietl, U Kämmerer

**Affiliations:** 1 Dept. of Obstetrics and Gynecology, University of Wuerzburg, Germany; 2 Dept. of Pathology, University of Wuerzburg, Germany

## Abstract

**Background:**

Tartrate-resistant acid phosphatase (TRAP) is a metalloprotein enzyme that belongs to the acid phosphatases and is known to be expressed by osteoclasts. It has already been investigated as a marker of bone metastases in cancer patients. In this study, which examined the value of serum TRAP concentrations as a marker of bone disease in breast cancer patients, we observed high concentrations of TRAP even in patients without bone metastases. To elucidate this phenomenon, we examined the expression of TRAP in breast cancer cells and the cells of several other malignancies.

**Methods:**

TRAP concentrations in the serum of tumor patients were determined by ELISA. The expression of TRAP in breast, ovarian, and cervical cancer and malignant melanoma was analyzed by immunohistochemistry. RT-PCR and immunocytology were used to evaluate TRAP expression in cultured tumor cells.

**Results:**

A marked increase in serum TRAP concentrations was observed in patients with breast and ovarian cancer, regardless of the presence or absence of bone disease. TRAP expression was found in breast and ovarian cancers and malignant melanoma, while cervical cancer showed only minimal expression of TRAP. Expression of TRAP was absent in benign tissue or was much less marked than in the corresponding malignant tissue. TRAP expression was also demonstrated in cultured primary cancer cells and in commercially available cell lines.

**Conclusion:**

Overexpression of TRAP was detected in the cells of various different tumors. TRAP might be useful as a marker of progression of malignant disease. It could also be a potential target for future cancer therapies.

## Background

Tartrate-resistant acid phosphatase (TRAP) is a metalloprotein enzyme belonging to the family of acid phosphatases, which are specifically grouped together because of their ability to catalyze the hydrolysis of orthophosphate monoesters under acidic conditions. TRAP has been studied extensively as a marker of bone disease in cancer patients [1-6]. It is normally found at low concentrations in the peripheral blood [7]. An increase in osteoclast activity is accompanied by synthesis and secretion of TRAP and serum TRAP activity is enhanced in the presence of increased bone resorption [8]. Elevated TRAP concentrations are found under pathological conditions such as Paget's disease, primary and secondary hyperparathyroidism, severe osteoporosis, and multiple myeloma, and in the presence of bone metastases originating from breast cancer and other malignancies [9,10].

These observations suggest that acid phosphatases might be diagnostically useful as serological and histological markers of progression and that they might also be of use in the investigation of the corresponding pathophysiological process. TRAP has been used as a diagnostic marker for hairy cell leukemia for more than two decades [1]. To clarify the significance of this enzyme in solid tumors, we investigated serum TRAP concentrations in patients with breast and ovarian cancers and the expression of TRAP in the primary tumors of patients with breast, ovarian or cervical cancer or malignant melanoma, and cultured cells from effusions/ascites associated with such tumors. Expression of the TRAP protein or mRNA were evaluated, rather than TRAP activity.

## Methods

### Ethics

The experimental research and the collection of patients' cells, tissue, and serum for the study was approved by the Ethics Committee of the Medical Faculty of the University of Wuerzburg, Germany, reference number 139/00. All patients participating in the study gave written informed consent.

#### 1. Serum and tissue samples

Serum samples were collected from patients with breast (n = 34) and ovarian (n = 39) cancer and from 37 controls, of whom 17 were premenopausal and 20 were postmenopausal (Table 1). A history of medications or conditions that could possibly affect bone metabolism (e.g. osteoporosis, thyroid disease, fractures, rheumatoid arthritis, osteoarthritis, steroid treatment) resulted in exclusion from the control group. Biopsy specimens of breast (n = 21), ovarian (n = 8) and cervical (n = 7) cancer, malignant melanoma (n = 6), benign breast tissue (n = 2), normal ovarian tissue (n = 3), and normal skin (n = 3) were investigated for TRAP expression by immunohistochemistry. 13 of the 34 breast cancer patients, but none of the ovarian cancer patients, had metastatic bone disease.

#### 2. Cell cultures

Primary tumor cells from malignant pleural effusions and ascites associated with metastatic breast cancer (4 cases) and ovarian cancer (4 cases) were isolated and cultured. The effusions had been aspirated because of clinical symptoms. The aspirates (20–500 ml) were centrifuged, and the resulting cell pellets washed twice in phosphate buffered saline (PBS, Biochrom, Berlin, Germany). Cells were cultured in HBCA medium [12] supplemented with 10% fetal bovine serum (FBS, PAA Laboratories, Cölbe, Germany) and gentamycin (50 μg/ml, Biochrom) at 2 × 10^5 ^cells/ml in a plastic cell-culture flask in a humidified incubator (atmosphere of 5% CO_2_). Contaminating fibroblasts were eliminated by trypsin treatment every other day and the remaining tumor cell monolayer was cultured until the cells were of homogeneous morphology (passage 3–4). If the tumor cells had divided adequately, contaminating leukocytes and fibroblasts were absent after these few passages.

The following commercially available cell lines were also investigated: Breast cancer cell lines MCF7, MDA-MB-468, BT20, and HBL-100, ovarian cancer cell lines OAW42 and SKOV3, and cervical cancer cell lines SiHa and CaSki. The breast and ovarian cancer cell lines were obtained from Cell Line Services (Heidelberg, Germany) and cervical cancer cell lines from the Department of Obstetrics and Gynecology, University of Jena, Germany. All cell lines were cultured in RPMI1640 medium (Biochrom) supplemented with 10% FBS and gentamycin (Biochrom).

#### 3. Enzyme-Linked Immunosorbent Assay (ELISA)

The serum samples were analyzed using a TRAP-ELISA Kit (Medac, Wedel, Germany) according to the manufacturer's instructions. The range of the ELISA was 1–10 U/l. In some cases the TRAP concentration was higher than 10 U/l: these samples were diluted 1:4 in the sample dilution buffer provided with the kit and reanalyzed. Final serum concentrations were then derived by multiplication of the value obtained by four.

#### 4. Immunohistochemistry and immunocytology

Immunocytological investigations were performed on the cells cultured from the malignant pleural effusions and ascites and commercially available established cell lines. Cells were seeded onto APES-(3-amino-propyltriethoxy-silane; Roth, Karlsruhe, Germany) coated slides and grown over night. They were then fixed with 4% formalin and washed in PBS for 10 min, followed by distilled water for 2 minutes and then acetone/methanol [1:2 for 5 min].

For immunohistochemistry, sections of routinely-processed paraffin-embedded tissue were cut at 2–3 μm, placed onto APES-coated slides, dewaxed in xylene, and rehydrated in graded ethanols and TRIS-buffered saline (TBS; 25 mM TRIS/HCl, pH 7.4, 137 mM NaCl, 2.7 mM KCl). For antigen retrieval, sections were subjected to heat pretreatment by boiling in 0.01 M sodium citrate buffer (pH 6.0) for 10 min in a microwave oven (600 Watt/sec.).

For specific detection of the TRAP antigen, the sections and cell smears were incubated with goat serum for 30 min and then with the TRAP monoclonal antibody at 1:100 dilution in antibody diluent (DAKO, Hamburg, Germany). The TRAP antibody is directed against the N-terminal portion of human TRAP (clone: 26E5, subtype: IgG2b; Novocastra Laboratories Ltd., Newcastle, United Kingdom). After one wash in PBS, the horseradish-peroxidase (HRP)-labeled rabbit anti-mouse specific secondary antibody (DAKO, dilution 1:100) was applied. The detection reaction was developed with 3,3'-diaminobenzidine (DAB; Sigma, Deisenhofen, Germany) or Vector VIP (Vector Laboratories, Burlingame, CA, USA). The sections and smears were counterstained with hematoxylin (Mayers, Sigma), dehydrated through graded ethanols, and embedded in Entelan (Merck, Darmstadt, Germany).

Immunoreactivity of the cells and tissues was evaluated by comparison with control sections incubated with an IgG control antibody. They were rated positive [+] if a clear brown or purple color was detected in the majority of the cells, as weakly positive [(+)] if less than 50% of the cells were stained (the majority weakly)), and as negative [-] if no staining was detectable.

#### 5. RT-PCR

RNA was extracted from the cell lines obtained from the patients and from the commercial cell lines using the Quiagen RNA-extraction kit (Quiagen, Hamburg, Germany). LPS-activated dendritic cells, which have been described to express TRAP [13], and JAR choriocarcinoma cells served as negative controls, respectively. mRNA was transcribed with reverse transcriptase and oligo T-primers (both from Promega, Mannheim, Germany). 5 μg of cDNA were used for PCR. Two different sets of primers, which were designed specifically to amplify mRNA of human TRAP 5b, were used. Both gave identical results. The first set had the following sequences: upstream-primer 3'CTTTCTACCGCCTGCACTTC5', and downstream primer 3'GCTGTTTCTTGAGCCAGGAC5'. The second primer pair consisted of the upstream primer 3'AGGCTTTTCCTCCAACCTGT5' and downstream primer: 3'GGAACTCAGCAAAGGTGAGC5', Taq polymerase (Promega) was used at 1 U/μl in the buffer supplied by the manufacturer with 5 mM dNTP and 25 mM MgCl_2_. The PCR was run for 40 cycles and the temperatures were 96°C, 60°C (annealing), and 72°C. The PCR products corresponding to the mRNA were 171 and 175 base pairs in size for the two different primer pairs respectively. PCR products were run on a 1% agarose gel and analyzed. RT-PCR was considered positive when a clear band of the expected size was visible on an ethidium bromide-stained gel.

## Results

### 1. Enzyme-Linked Immunosorbent Assay(ELISA)

#### TRAP 5b in the serum of tumor patients and controls

**Figure 1:**
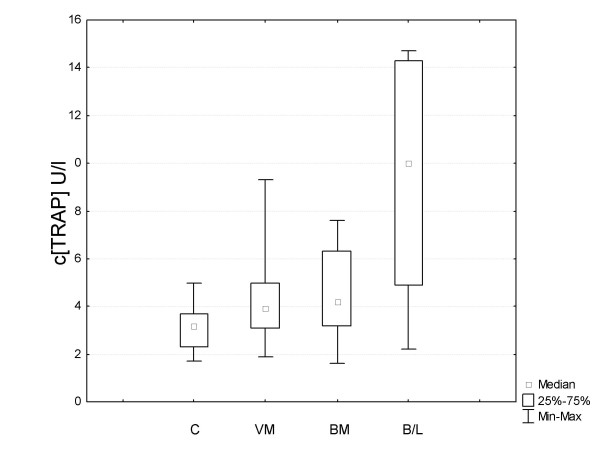
Analysis of serum of breast cancer patients and controls by the ELISA technique. Box and whisker blot of ELISA results from patients' serum. C = total control group, VM = visceral metastases, BM = bone metastases, B/V = bone and visceral metastases.

The median values for serum TRAP concentrations are summarized in Table 1 and Figure 1. Serum from breast and ovarian cancer patients as well as controls were analyzed by ELISA. The TRAP concentration was above the detection limit in all the samples analyzed. Four of the samples from patients with visceral and bone metastases showed concentrations above the upper standard and the final concentration was therefore calculated from diluted samples.

We first evaluated serum from 14 breast cancer patients who had visceral metastases only, and found increased values (median 4.0) in almost all of these patients as compared to the control group as a whole (median 3.2) (Table 1). The values were comparable to those in patients with metastases confined to the bones. Interestingly, the highest serum concentrations were found in patients with aggressive disease, with both bone and visceral metastases (median 10) (Table 1).

Serum TRAP protein concentrations in 40 ovarian cancer patients with advanced disease were also determined. Analysis revealed that there were essentially two groups of ovarian cancer patients: one with high serum TRAP concentrations (median 3.7), almost equalling those of breast cancer patients with bone disease, and a second group with very low values (median 1.9).

### 2. Immunohistochemistry

We tested various malignant and benign tissues for the expression of TRAP (Table 2). The majority of the breast cancer specimens (15 out of 21), ovarian cancer specimens (6 out of 8) and malignant melanoma specimens (5 out of 6) were found to express TRAP, whereas the corresponding benign tissue showed markedly weaker expression or none at all. Only one of the 7 cervical cancer specimens showed TRAP expression.

**Figure 2:**
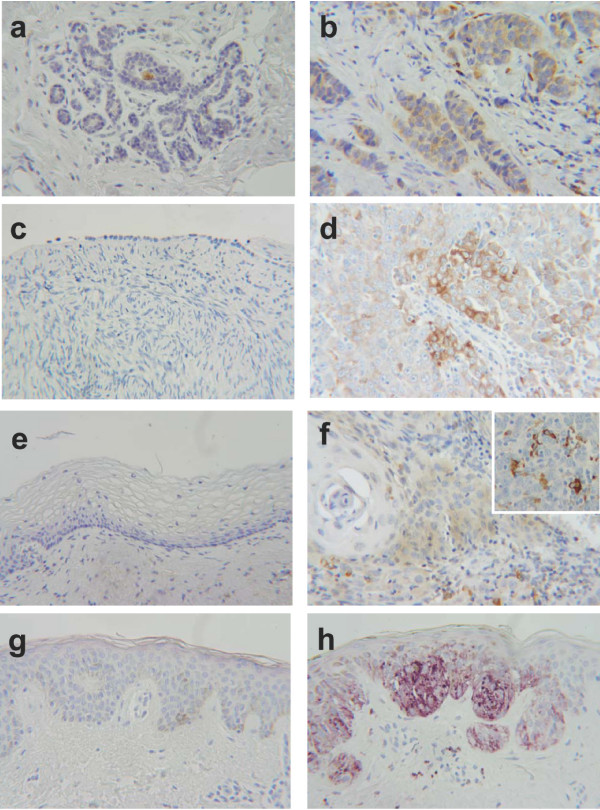
Immunohistochemical findings in breast, ovarian, and cervical cancer and malignant melanoma, and corresponding benign tissue. (magnification 250×) a) Normal breast tissue with no detectable expression of the TRAP protein by immunohistochemistry. The brown staining in the duct is due to nonspecific staining of secretions. b) Invasive ductal carcinoma of the breast with significant expression of TRAP protein. (brown = DAB) c) Benign ovarian celomic epithelium with no expression of TRAP. d) Serous papillary ovarian cancer cells expressing TRAP (brown = DAB). e) No TRAP expression is detectable in non-transformed cervical tissue. f) Only weak expression of TRAP is seen in this cervical cancer specimen. Tumor-infiltrating monocytes clearly stain for TRAP, as shown in the enlarged section (brown = DAB). g) No expression of TRAP is detectable by immunohistochemistry in normal skin (VIP). The slight brown staining is due to skin pigmentation. h) There is marked overexpression of TRAP in the malignant melanoma in the same specimen as in (g), allowing differentiation of the malignant tumor from the adjacent normal skin. The slight brown staining is due to skin pigmentation. (purple = VIP)

Figure 2 illutstrates typical immunohistochemical findings. In contrast to normal tissue, the breast cancer specimen clearly stained for TRAP (Figures 2a and 2b). Likewise in serous ovarian carcinoma (Figure 2d), most tumor cells expressed TRAP, whereas normal celomic ovarian epithelium was negative (Figure 2c). Figure 2f shows the only TRAP-positive cervical cancer specimen. Interestingly, all the cervical cancer specimens exhibited pronounced infiltration by TRAP-expressing monocytes (Figure 2f). No TRAP expression could be detected in normal skin, whereas malignant melanoma cells showed very strong expression of TRAP (Figures 2g and 2h).

### 3. Immunocytology

**Figure 3:**
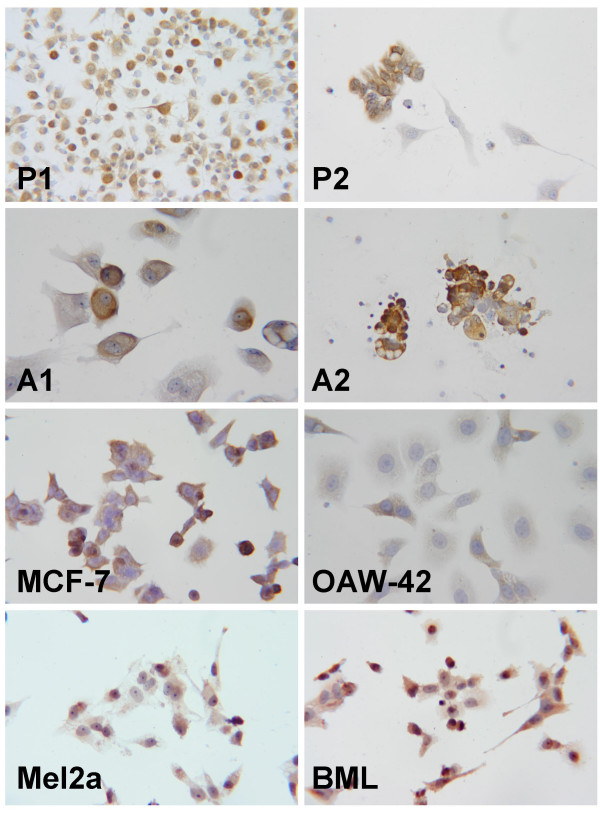
Immunocytology of cells cultured from pleural effusions and malignant ascites (magnification 400×). a) Cells cultured from a malignant pleural effusion due to metastatic breast cancer. Staining of the cytoplasm for TRAP is seen. P1 b) Cells cultured from a malignant pleural effusion due to metastatic breast cancer. with marked expressison of TRAP in most of the cells. P2 c) Cells cultured from malignant ascites in a patient with ovarian cancer with obvious staining for TRAP. A1 d) Cells cultured from malignant ascites in a patient with ovarian cancer demonstrating staining for TRAP in the cytoplasm. A2 e) The commercially available cell line MCF 7 with staining for TRAP. f) The established cell line OAW42 also shows staining for TRAP. g) The malignant melanoma cell line Mel2A with moderate but unequivocal staining for TRAP. h) Malignant melanoma cell line BML with staining for TRAP.

The immunocytological results are summarized in Table 3 and illustrated in Figure 3. Cells cultured from 3 of the 5 malignant pleural effusions in breast cancer patients expressed TRAP abundantly. The established breast cancer cell lines MCF 7, MDA-MB-468, HBL-100, and BT20 were all TRAP positive. Cells cultured from the malignant ascites of 9 ovarian cancer patients were investigated after 3 to 4 in vitro passages. Five of the cultured cell lines were TRAP positive. Two established ovarian cancer cell lines, OAW42 and SKOV3, were also tested and found to express TRAP, although very weakly.

The malignant melanoma cell lines MV3, BML, and Mel2A showed marked expression of the TRAP protein, staining of the BML cells being stronger than that of the other two cell lines. Of the two cervical cancer cell lines investigated, SiHA cells showed weak staining for TRAP, but CaSki cells were negative (data not shown). Of the two choriocarcinoma cell lines JEG-3 was positive, but JAR was negative, so that they were used in the RT-PCR experiments as positive and negative controls, respectively.

### 4. RT-PCR

**Figure 4:**
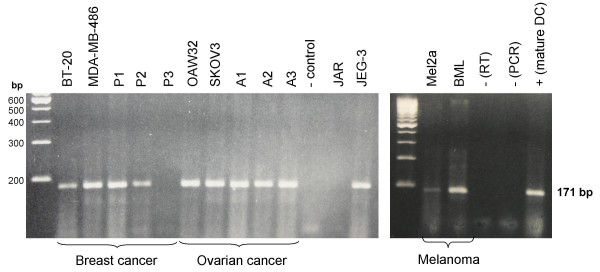
(*Large gel, left side*) TRAP mRNA expression in commercially available cell lines and cultured cells from malignant effusions, as detected by RT-PCR. The breast cancer cell lines BT20 and MDA-MB-468, and 2 (P1 and P2) of 3 cell cultures derived from breast cancer patients with malignant pleural effusions show TRAP mRNA expression, whereas no TRAP mRNA is detectable in cells from patient P3. Ovarian cancer cell lines OAW42 and SKOV3 and cells cultured from the malignant ascites of ovarian cancer patients A1 – A3 show TRAP mRNA expression. No TRAP mRNA is detectable in the choriocarcinoma cell line JAR, unlike choriocarcinoma cell line JEG-3, which shows marked expression. (*Small gel, right side*) Detection of TRAP mRNA expression in malignant melanoma and stimulated dendritic cells by RT-PCR. Malignant melanoma cells BML and Mel2a show expression of TRAP mRNA, expression being stronger in the latter. As expected, stimulated dendritic cells show strong expression of TRAP mRNA.

In order to confirm TRAP expression in the tumor cells with another method of detection, RT-PCR analysis of TRAP mRNA levels in the cell cultures under investigation was performed. (Figure 4).

Strong expression of TRAP mRNA was found in the two breast cancer cell lines tested (BT20 and MDA-MB-468) and in breast cancer cells cultured from pleural effusions in 2 of the 3 patients investigated. The commercially available cell lines OAW42 and SKOV3 and ovarian cancer cells cultured from three patients with malignant ascites showed a clear PCR product at 171 bp. The two malignant melanoma cell lines BML and Mel2A both showed TRAP mRNA expression, which was more pronounced in the former than in the latter. Repeat RT-PCR experiments consistently revealed TRAP mRNA expression in JEG-3 cells but none in JAR cells.

## Discussion

In contrast to previous studies [1-3], our investigations revealed high serum concentrations of TRAP not only in breast cancer patients with bone metastases but also in such patients with visceral metastases alone.

Our immunohistochemical and immunocytological studies showed that a broad range of tumor tissues and cells express TRAP. From our results we conclude that TRAP is overexpressed in a variety of solid tumors and is most probably secreted by the tumor cells themselves. The finding that cancer cells express and secrete TRAP is not yet well established. Therefore, we evaluated this finding not only on the mRNA but also on the protein level.

TRAP expression was detected by immunohistochemistry and RT-PCR in commercially available cell lines and cells cultured from malignant pleural effusions in breast cancer patients and in biopsy specimen of breast cancer. Approximately half of the ovarian carcinoma cell cultures derived from malignant ascites and surgical specimens of ovarian cancer were positive for TRAP. Likewise, both malignant melanoma tissue and cell lines clearly expressed TRAP. On the other hand, TRAP could not be detected in cervical cancer tissue. TRAP was not detected in the malignant cells of primary carcinomas of the cervix, but these tumors showed marked infiltration by TRAP-expressing monocytes.

TRAP expression seems to be confined to malignant tissue, as it was not detected in normal breast or ovarian tissue or normal skin. It appears to be a basic characteristic of malignant transformation, as it is found in both epithelial and mesenchymal malignancies, such as the malignant melanoma investigated.

TRAP has recently been analyzed in cell cultures and was detected in both the supernatant medium and cell lysates [14]. Janckila et al. (2005) demonstrated that both isoforms of TRAP are secreted into the supernatant, which suggests that the high serum values found in our investigation originate from secretion by tumor cells [14]. In contrast to these authors we did not use cultured cells of the moncytic lineage to evaluate TRAP levels, but primary cancer cells and established cell lines from several malignant tumors [14].

Our results are consistent with the recent findings of Chao et al. (2005), who describe a significant increase in TRAP in the serum of breast cancer patients with extensive bone metastases [2]. It can be assumed that the majority of breast cancer patients with very extensive bone metastases also have visceral involvement. In the investigation of Chao and coworkers, patients with limited bone disease were not found to show increased TRAP concentrations. In addition, their TRAP concentrations were lower than in patients with visceral metastases alone, although this finding was not statistically significant [2]. This lack of statistical significance is probably due to the small number of patients with visceral metastases and no bone involvement. Nguyen et al. (1991) described an increase in serum TRAP values in patients with metastatic breast cancer [4]. Interestingly, this increase was independent of the presence of bone metastases. This finding can now be explained by our results.

The cellular function of TRAP and the biological significance of its expression in physiological conditions such as pregnancy or pathological states such as Gaucher's disease are still not fully understood [15-17]. Our results suggest that TRAP could serve as a marker of progressive disease, rather than being a selective marker of bone metastasis.

Future studies may be able to determine whether serum TRAP concentrations can be used to monitor the success of chemotherapy. Even more interesting is the possibility that TRAP could be an attractive target for future cancer therapies, given its predominant expression in malignant tissue.

## Competing interests

The author(s) declare that they have no competing interests.

## Authors' contributions

AH drafted the manuscript, recruited patients, designed and coordinated the study, LR participated in its design and coordination, MK carried out the immunohistochemical and ELISA studies, MKr carried out the molecular studies, ME evaluated the immunohistochemical data, JD participated in the design of the study and patient recruitment UK participated in the design of the study, supervised the laboratory work, evaluated immunohistochemical data and performed the statistical analysis. All authors read and approved the final manuscript.

## Pre-publication history

The pre-publication history for this paper can be accessed here:



## Figures and Tables

**Table 1:** Median TRAP -concentrations in serum

**diagnosis**	**no. of patients**	**metastases**	**median c [TRAP] U/l**
breast cancer	7	bone and visceral	**10**
breast cancer	7	only liver	5.0
breast cancer	6	only bone	4.1
breast cancer	14	only visceral	4.0
ovarian cancer I	13	visceral	3.7
ovarian cancer II	26	visceral	1.9
			
control pre-menopausal	17	-	2.6
control post-menopausal	20	-	3.4
total control group	37	-	3.2

**Table 2:** Immunohistochemical evaluation of TRAP expression in malignant and benign tissue samples.

	**tissue sample**	**no. of samples**	**no. of TRAP- positive samples**
malignant	breast cancer	21	15
	ovarian cancer	8	6
	cervical cancer	7	1
	malignant melanoma	6	5
			
benign	normal breast tissue	2	0
	normal ovarian tissue	3	0
	normal skin	3	0

**Table 3:** Immunocytology of commercially available cancer cell lines and cells cultured from malignant pleural effusions and malignant ascites. TRAP staining was classified as follows: + positive, (+) weakly positive, - negative.

**source**	**cell line**	**TRAP expression**
breast cancer cell lines	MDA-MB-468	+
	MCF-7	+
	BT20	+
	HBL-100	+
primary breast cancer cells	P1	+
	P2	+
	P3	-
ovarian cancer cell lines	OAW42	(+)
	SKOV3	(+)
primary ovarian cancer cells	A1	+
	A2	+
	A3	+
cervical cancer cell lines	SiHa	(+)
	CaSki	-
melanoma cell lines	BML	+
	Mel2A	(+)
	MV3	+
